# After the Pandemic: The Future of Italian Medicine. The Psychological Impact of COVID-19 on Medical and Other Healthcare-Related Degrees Students

**DOI:** 10.3389/fpsyg.2021.648419

**Published:** 2021-11-04

**Authors:** Giacomo De Micheli, Laura Vergani, Davide Mazzoni, Giulia Marton

**Affiliations:** ^1^Vita-Salute San Raffaele University, Milan, Italy; ^2^Department of Oncology and Hemato-Oncology, University of Milan, Milan, Italy; ^3^Applied Research Division for Cognitive and Psychological Science, European Institute of Oncology (IRCCS), Milan, Italy

**Keywords:** students, health-care professionals, COVID-19, psychological symptoms, pandemic, emotional reactions

## Abstract

**Objective:** The study aimed to explore the psychological symptoms and the readiness to fight the pandemic of the new generation of healthcare professionals: medical and other healthcare degree students.

**Methods:** We enrolled 509 medical and healthcare-related degree students during the second outbreak of COVID-19 in Italy. We have examined their psychological symptoms using the 12-item General Health Questionnaire (GHQ-12) and their readiness to fight the pandemic together with their academic career status, their relationship with the university, and their emotional reactions to the pandemic with Visual Analog Scales.

**Results:** We retrieved a GHQ mean of 21.65 (SD = 40.63) and readiness to fight the pandemic mean of 53.58 (SD = 31.49). Perceived control affects variables: a negative effect on psychological symptoms and a positive effect on the willingness to fight the pandemic. The other variables with an impact were stress, loneliness, and anger that had a significant and positive impact on psychological symptoms. Age and concern for patients had a significant positive impact on readiness to fight for the pandemic, while years of attendance had a significant but negative impact.

**Conclusion:** Universities and Institutions should consider the impact of the pandemic on students, in particular, for its effect on their mental health.

## Introduction

The COVID-19 pandemic has been affecting the lives of people significantly for the past year. Italy, in particular, has been one of the most affected countries, with a substantial number of cases that, despite numerous restrictions, stopped increasing for a while (Distante et al., 2020; Remuzzi and Remuzzi, 2020).

During this tough time, we have experienced an apocalyptic-like scenario where the streets were empty and the hospitals were full. People were forced into their homes starting from March 9, 2020, to May 18, 2020, with this affecting significantly their quality of life and mental health status (Masiero et al., 2020; Pizzoli et al., 2020; Prete et al., 2020; Serafini et al., 2020; Monzani et al., 2021).

Initially, the Italian Government tried to loosen the containment measures, since the cases seemed to decrease, but unfortunately, the situation resumed quickly, after a few months from the end of the first phase (Bontempi, 2020; Wise, 2020).

Hospitalization, infections, and deaths linked to COVID-19 returned and rose exponentially, leaving people facing new restrictions and several hard consequences: freedom of movement was severely constrained in most of the Italian regions, the majority of workers started to operate from home again, shops were closed, and restaurants were forced to limited opening, with this having a major impact on the economy.

In hospitals, the situation became critical, leading to the suspension of non-urgent procedures and wards converted for COVID-19, therefore, affecting the quality of life of those who needed treatment and putting even more stress on healthcare workers.

Starting from March 5, face-to-face educational activities of the academic year 2019–2020 were suspended in both schools and universities throughout the country to limit the spread of the virus. To deal with this situation, distance learning through various online platforms was initiated (Favale et al., 2020). Undoubtedly, this modality had several advantages. There is no need to share a room for hours, reducing the risks of infection (Saddik et al., 2020). On the other hand, there was a significant reduction of interaction between students and professors, with a loss of practical training opportunities. The mental health of the students was also brought into question.

In this regard, some studies were conducted with college students worldwide, demonstrating a strong emotional impact of the COVID-19 pandemic (Nurunnabi et al., 2020; Sundarasen et al., 2020; Batais et al., 2021; Pedraz-Petrozzi et al., 2021). For example, in a study conducted on Malaysian college students, the levels of anxiety during the peak of the COVID-19 crisis were high, and the major causes of stress were financial restrictions, distance learning, and uncertainty about the future, in particular, related to academic performance (Sundarasen et al., 2020). In China, students showed clinically relevant symptoms of anxiety, depression, and post-traumatic stress disorder (PTSD), with various factors making a contribution: for example, older age was linked with PTSD symptoms (Chi et al., 2020). In a recent study with Italian college students, depressive symptoms worsened during lockdown compared with 6 months before isolation (Meda et al., 2021). Multiple stressors were identified that contributed to the increased levels of stress, anxiety, and depressive thoughts, such as both factors related to university life and other emotional reactions to the situation (Son et al., 2020).

Among the college students, the worries of students in health professions were particularly enhanced by the situation, and this was true both because of the impact of the pandemic on their education and because of their possible commitment to fight the pandemic (Biavardi, 2020; Chandratre, 2020; Ferrel and Ryan, 2020).

Students in health professions are usually and generally more prone to stress, as demonstrated by some studies: they suffer from stress and burnout, and even higher rates of depression than the general population, with rates of women higher than rates of men (Dahlin et al., 2005; Watson et al., 2008). During the pandemic, studies conducted in different countries highlighted that medical students showed symptoms of depression, stress, or anxiety (Saddik et al., 2020; Batais et al., 2021; Nishimura et al., 2021; Pedraz-Petrozzi et al., 2021; Seetan et al., 2021). Among the factors associated with higher stress levels, studies identified female gender, worries about family, or personal health, and also, online learning (O’Byrne et al., 2020a). Nursing students were found to suffer from anxiety, stress, and fear (Temiz, 2020; Aslan and Pekince, 2021; Medina Fernández et al., 2021). In the healthcare students sample, smoking, using medications, and having lower family income are factors associated with higher anxiety during the COVID-19 pandemic (Basheti et al., 2021).

Consistent with these results, a recent study showed that the mental health of Italian healthcare workers was affected by the COVID-19 pandemic (Marton et al., 2020), suggesting that perceived control, concern for patients and family, feeling alone, and anger were significant predicting factors.

In this context, it was particularly interesting to study the perceptions of students of being able to fight the pandemic (O’Byrne et al., 2020b). This idea is compatible with studies of resilience and self-efficacy (Bandura, 1994) and with the more recent studies on perceived work readiness (Caballero and Walker, 2010). Much of the literature that has investigated the transition experiences of graduate healthcare workers have mostly focused on the stressors associated with the first years of work (e.g., Daly and Willcock, 2002; Willcock, 2005; Kelly and Ahern, 2009; Watson et al., 2009). However, less is known about the perceived efficacy to work in the context of the pandemic. In past pandemics, other studies had highlighted the willingness of medical students to be directly involved (Mortelmans et al., 2009; Waight et al., 2011), but little is known about factors associated with this desire: we may suppose that older students attending the last years of their university health professional education programs are more experienced, have more perceived efficacy, and therefore, are more ready to fight the pandemic. However, as noted, mental stress and other emotional reactions are common for students during this situation and may affect their readiness to fight the pandemic and the relationship they have with the university.

The aim of this study was to explore the mental health and readiness to fight the pandemic during the second wave of the COVID-19 pandemic. Based on previous studies conducted during similar events on college students and healthcare professionals (Chi et al., 2020; Marton et al., 2020; Son et al., 2020), we hypothesized increased psychological symptoms in students and different degrees of readiness to fight the pandemic on the basis of their current academic career and their emotional reactions to the COVID-19 pandemic. More specifically, we hypothesized that psychological symptoms of students and readiness to fight the pandemic were dependent on a number of factors, such as the academic career status (age and years of attendance), relationship with the university (feeling abandoned by the university, possible regret for the chosen program), and other emotional reactions to the pandemic (stress, feeling alone, anger, fear for patients, and perceived control).

## Materials and Methods

We performed an observational study with a sample of 509 students enrolled in healthcare programs during the second wave of COVID-19 in Italy. Recruitment started on November 23, 2020, and ended on December 5, 2020; it occurred through social media, mailing lists of universities, and snowball recruitment. The research was approved by the European Institute of Oncology Ethics Committee. Written informed consent was provided by the participants before they started to complete an online survey, which aimed to measure their mental health status and their response to the second wave of COVID-19 in Italy. In the introduction to the questionnaire, we explained that we wanted to assess how the emergency affected the lives of the students in terms of psychological symptoms, readiness to fight the pandemic, and their academic career status, relationship with the university, and other emotional reactions to the pandemic.

The online questionnaire was designed following the 12-item General Health Questionnaire (GHQ) Italian Translation (Piccinelli et al., 1993; Goldberg et al., 1997) and used Visual Analog Scales (VAS).

Specifically, the GHQ-12 assessed psychological symptoms. Examples of items were “have you felt unhappy or depressed?” and “have you lost confidence in yourself?” Four-point Likert scale answers were used in each question. Scores above the threshold of 13/14 are an indicator of the presence of psychological symptoms (Piccinelli et al., 1993; Goldberg et al., 1997).

Fourteen VAS were designed by authors to explore the readiness of students to fight the pandemic, their academic career status, their relationship with the university, and their emotional reactions to the pandemic. Examples of items were “You are feeling stressed,” “You are feeling in control of the situation in everyday life,” “You are feeling lonely,” and “If you chose a career in healthcare, how ready would you feel to face the current pandemic situation.” The VAS had a range from 0 (not at all) to 100 (completely), with high values indicating the worse condition except for questions concerning the compliance with the rules, the perceived control on the situation, and readiness to respond to it. The full VAS are reported as Supplementary Material.

Using SPSS 26, we performed descriptive statistics (mean and SD), bivariate correlation, and multivariate regression analysis, considering *p* < 0.05 as a statistical significance.

## Results

The sample was composed of 362 (71.1%) medical students, 132 (25.9%) nursing students, and 15 (2.9%) students of other healthcare professions. Among the participants, 374 (73.5%) were female and 133 (26.1%) male; two (0.4%) persons preferred not to specify their gender.

Regarding the year of attendance, 166 (32.6%) participants were attending the first year, 213 (41.8%) the second, 66 (13.0%) the third, 27 (5.3%) the fourth, one student (0.2%) attending the fifth, and 36 (7.1%) students the sixth. In the sample, 418 (82.1%) students were not doing clinical internships whereas 91 of them (17.9%) were doing.

Since restrictions varied from region to region, we decided to consider the areas from which the participants came: 33 participants (6.5%) were located in “yellow areas,” (low risk), 248 (48.7%) in “orange areas” (moderate risk), and 228 (44.8%) in “red areas” (high risk) according to the regions risk scenarios established by the Italian Government (Decree by the President of the Council of Ministers on November 3, 2020, published in the Italian official journal no. 275).

### Descriptive Statistics

Descriptive statistics of our study variables can be found in Table 1.

**TABLE 1:** Descriptive statistics of study variables.

	Minimum	Maximum	Mean	SD
Psychological symptoms	5	33	21.65	4.63
Readiness to fight the pandemic	0	100	53.58	31.49
Age	18	46	21.28	3.08
Year of attendance	1	6	2.19	1.33
Feeling abandoned by university	0	100	40.35	33.92
Regret for the chosen degree	0	100	6.17	16.86
Stress	0	100	65.22	24.92
Loneliness	0	100	51.43	34.25
Anger	0	100	45.61	33.24
Fear for their patients	0	100	27.09	36.85
Perceived control	0	100	48.16	27.52
Fear for themselves	0	100	33.65	27.07
Fear for family member/cohabitant	0	100	71.35	28.39
Feeling worried about the effect of the pandemic on the timing of their degree	0	100	30.89	35.12
Respect for the rules imposed by the government	0	100	90.50	17.26
Undertaken preventative behavior	0	100	87.86	16.62
Stress related to the region	0	100	60.40	31.33

In particular, regarding the key variables, the mean of psychological symptoms was 21.65 (SD = 4.63) above the threshold of 13/14, therefore, indicating a high level of distress. The VAS investigating the readiness to fight the pandemic had a mean of 53.58 (SD = 31.49). Regarding academic career status, we retrieved a mean age of 21.28 (SD = 3.08) and a mean year of attendance of 2.19 (SD = 1.33). VAS investigating the relationship with the university—feeling abandoned by the university and possible regret for the chosen degree—had respectively, a mean of 40.35 (SD = 33.92) and 6.17 (SD = 16.86). VAS related to emotional reactions to the pandemic included stress (mean = 65.22; SD = 24.92), loneliness (mean = 51.43; SD = 34.25), anger (mean = 45.61; SD = 33.24), fear for patients (mean = 27.09; SD = 36.85), and perceived control (mean = 48.16; SD = 27.52).

### Correlations and Regression Analyses

Results of the correlational analysis between the key variables are reported in Table 2.

**TABLE 2:** Correlations between the key variables.

	Variables	1	2	3	4	5	6	7	8	9	10	11
1	Psychological symptoms		−0.09*	−0.07	−0.07	0.21**	0.15**	0.53**	0.43**	0.36**	−0.04	−0.38**
2	Readiness to fight the pandemic			0.15**	−0.19**	−0.08	−0.05	−0.06	−0.06	0.00	0.23**	0.18**
3	Age				0.37**	0.2*	0.05	−0.09*	−0.12**	−0.1*	0.16**	0.16**
4	Years of attendance					0.32**	0.09*	−0.11*	−0.08	−0.16**	0.08	0.07
5	Abandoned by university						0.2**	0.21**	0.20**	0.21**	0.02	−0.07
6	Regret for the chosen degree							0.05	0.05	0.07	0.06	−0.15**
7	Stress								0.37**	0.43**	0.03	−0.28**
8	Loneliness									0.38**	0.06	−0.24**
9	Anger										0.02	−0.2**
10	Fear for their patients											−0.03
11	Perceived control											

*Significant correlations 0.05 two-tailed; **significant correlations 0.01 two-tailed.

We then performed a multivariate regression analysis, considering psychological symptoms and readiness to fight the pandemic as dependent variables whereas academic career status variables (i.e., age and years of attendance), relationship with the university variables (i.e., feeling abandoned by the University, possible regret for the chosen degree), and emotional reactions to the pandemic variables (i.e., stress, loneliness, anger, fear for patients, and perceived control) were regarded as independent.

We considered partial η^2^ as an effect size measure, following the classification of Cohen (1988): 0.01, as small effects, 0.06 as medium effects, and 0.14 as large effects.

Variance inflation factor (VIF) indicators were considered to verify that multicollinearity did not represent a problem.

Detailed results and coefficient values of the multivariate regression analysis are reported in Table 3.

**TABLE 3:** Results and coefficients of multivariate regression analysis.

Dependent variable		B	*p*	95% C.I.		Partial η^2^
Psychological symptoms	Age	0.085	0.307	−0.078	0.247	0.004
	Year of attendance	−0.036	0.841	−0.388	0.316	0.000
	Abandoned by university	0.009	0.208	−0.005	0.024	0.006
	Regret for the chosen degree	0.023	0.077	−0.002	0.048	0.011
	Stress	0.062	0.000	0.042	0.082	0.116
	Loneliness	0.026	0.000	0.012	0.040	0.044
	Anger	0.017	0.031	0.002	0.033	0.016
	Fear for their patients	−0.011	0.068	−0.023	0.001	0.012
	Perceived control	−0.031	0.000	−0.048	−0.015	0.046
Readiness to fight the pandemic	Age	2.335	0.000	1.114	3.557	0.048
	Year of attendance	−5.878	0.000	−8.519	−3.237	0.063
	Abandoned by university	−0.010	0.861	−0.119	0.100	0.000
	Regret for the chosen degree	−0.006	0.953	−0.194	0.183	0.000
	Stress	−0.059	0.441	−0.208	0.091	0.002
	Loneliness	−0.041	0.444	−0.147	0.064	0.002
	Anger	0.062	0.299	−0.055	0.179	0.004
	Fear for their patients	0.188	0.000	0.097	0.279	0.055
	Perceived control	0.211	0.001	0.087	0.335	0.038

We retrieved satisfactory *R*^2^ values for both psychological symptoms and readiness to fight the pandemic (respectively: *R*^2^ = 0.38 and adjusted *R*^2^ = 0.36; *R*^2^ = 0.19 and adjusted *R*^2^ = 0.16).

Perceived control was the only variable having a significant effect on both the two dependent variables: a negative effect on psychological symptoms and a positive effect on the readiness to fight the pandemic. Stress, loneliness, and anger had a significant and positive effect on psychological symptoms. Age and concern for patients had a significant positive effect on readiness to fight the pandemic, while years of attendance had a significant but negative effect.

## Discussion

Even though some opinion articles and editorials are circulating (e.g., Biavardi, 2020), to the best of our knowledge, this is the first study aiming to investigate psychological symptoms and readiness to fight the pandemic of a sample of students in health professions in Italy during the COVID-19 emergency, in particular, during the second wave.

In a world that is changing unexpectedly, in which healthcare professionals are pushed to fight the virus at the cost of their safety and lives (Marton et al., 2020), some students are looking at the healthcare crisis and at the difficulties of healthcare providers imagining their future and projecting themselves into it.

Overall, we found that students in health professions in our sample respected the rules imposed by the government to restrict the pandemic, and they think that their behavior is protective, but fear for their family members and cohabitants is high. We can only assume that following our previous results found in a healthcare professional sample fighting against COVID-19 in phase 1 of the Italian pandemic, once they start their jobs face to face with COVID-19-affected patients, the fear could grow together with their psychological distress (Marton et al., 2020). Their psychological symptom level was above the threshold of the GHQ-12 indicating a high level of distress. All of these factors contribute to making the condition of the students in health professions a difficult one, but they do not regret their choice in studying in a healthcare profession degree—the mean of the VAS investigating the feeling of regret for the chosen degree is low. This last result is in line with the findings of Bai et al. (2021) in a group of Chinese nursing students. The commitment of students to nursing as a profession increased after the beginning of the pandemic; the authors highlighted as possible reasons the positive image of nurses provided by media and the implementation of policies to improve the welfare of nurses during the pandemic.

The mean of the VAS investigating the readiness to fight the pandemic is moderately high, but it could be comparable to other similar results retrieved in different pandemic contexts. In the past H5N1 pandemic, 88.0% of medical students interviewed in Michigan expressed their desire to be part of the healthcare staff (Waight et al., 2011). Moreover, during the same pandemic, the vast majority of Belgian senior medical students were willing to “being involved in implementing primary care” and “would care for pandemic patients if necessary” (Mortelmans et al., 2009, 438). Despite their fears, for themselves and their families, their anger, and their high level of psychological symptoms, our sample of students would like to “take the field,” exposing themselves directly to the challenges of managing the COVID pandemic.

### Psychological Symptoms

The psychological symptoms of students in health professions are explained by four variables, all pertaining to their emotional reactions to the pandemic: stress, loneliness, anger, and perceived control. Distress is defined as “a state of emotional suffering associated with stressors and demands that are difficult to cope with in daily life” (Arvidsdotter et al., 2016, 687) and is associated with many negative emotions: distress could impact the emotional status by changing it (Ridner, 2004; Carmassi et al., 2020).

Previous literature indicates that psychological distress could be related to different variables. In a sample of Chinese health professional students, psychological distress experienced during the pandemic and symptoms of acute stress reaction have been linked to adverse childhood events, life stressors, and internet addiction (Li et al., 2021a). The pandemic of COVID-19 increases stress, loneliness, and anger in students in health professions (Wang et al., 2020). These negative emotions could play a major role in the first year of training (Pitkälä and Mäntyranta, 2004; Kremer et al., 2016).

Another factor that contributed to explaining psychological symptoms is perceived control of the situation. The data are in line with the results of other previous studies (e.g., Zheng et al., 2020), which found that life satisfaction and perceived general health during the COVID-19 pandemic are affected by perceived control of the situation, and they concluded that they could be considered as protective factors against the negative effects of the pandemic. Our results add some interesting nuances—perceived control also affects the distress of students in health professions.

These data, indicating the high level of distress of this population in this particular situation, could be useful to Institutions and Universities. Distress in college students is often linked to costly consequences—such as worsening academic achievement or risky behaviors (Sharp and Theiler, 2018). For these reasons, it could be useful to implement appropriate and tailored interventions to identify vulnerable subjects, support students, and prevent negative effects. These interventions could be focused on implementing coping strategies helpful to overcome this particular situation and future ones.

### Readiness to Fight the Pandemic

In our study, students reported that they were quite ready to fight the pandemic. This appears consistent with other studies in other national contexts, in which students indicated their willingness to join the healthcare response to the COVID-19 pandemic (Hong et al., 2021).

Moreover, we found that four variables participated in explaining the readiness to fight the pandemic. The variables, regarding academic career status, age, and year of attendance had the strongest effect.

In particular, the older students seemed to be more ready to fight the pandemic compared to younger students. To explain these results, we could borrow from Petersen (2020) the concept of “optimistic anxiety”: in order to face the pandemic optimally, individuals should be both anxious and optimistic, anxious that they follow the rules and optimistic enough to believe that what they do could make a difference. This concept addresses the general population, but we think that it could be translated into the situation of students in health professions. It may be that, older students are more experienced and they acknowledge that fear is a natural reaction when facing a pandemic, but they also know that fear should be addressed and that it becomes more manageable when acknowledged (Finset et al., 2020); they are “optimistically afraid” and they feel ready to fight the pandemic admitting their fears.

This result needs to be contrasted with the finding that years of attendance (differently from age) had a significant but negative effect on readiness to fight the pandemic: so, students with less academic experience feel more ready to fight the pandemic. Age and year of attendance are different variables: in particular, year of attendance refers to the academic year that students are enrolled in. For example, a 33-year-old student can be currently enrolled in the first year, along with a 21-year-old student. At the end of the 20th century, Kruger and Dunning (1999, 30) discovered that “people tend to hold overly favorable views of their abilities in many social and intellectual domains.” In fact, “inexperienced people often have a falsely elevated sense of confidence about their performance” (Rahmani, 2020, 532). Various studies have been conducted, especially with young physicians, highlighting this effect also in the medical field. We may cautiously hypothesize that this effect could account also for our results: students in health professions in their very first years of attendance, with limited knowledge and experience, may falsely feel more confident, and more ready to directly face the healthcare crisis.

None of the relationships with the university variables had a significant effect on the readiness to fight the pandemic.

Two of the emotional variables, instead, participated in explaining the feelings of the students on being ready to fight. Fear for patients had a significant and positive effect, while in most cases, students had to interrupt internships and clinical activities. Some students in health professions may continue their activities or may be involved in some volunteering services, having direct contact with patients. The fear for the survival of patients and their condition is one of the most important elements that push healthcare students in their willingness to be involved in the fight against COVID-19. Healthcare professions, indeed, are often characterized by “empathy, compassion, engagement, and a wish to be of use” (González, 2012, 52). The readiness to fight the pandemic is influenced also by other emotional reaction variables: the more perceived control, the more students are willing to directly face the health crisis. We think that this result is complementary to the previously explained effect that perceived control has on psychological symptoms. As already pointed out, perceived control could be considered as a sort of protective factor against the psychological and mental effects of the pandemic.

### Limitations

Our study is not exempt from some limitations. First of all, our sample is not perfectly representative of all the population of students in health professions in the Italian context: the vast majority, more than 70%, was composed of medical students, while only 30% students came from other healthcare-related degrees. The percentage of women in our sample (about 70%) reflects the real situation in the health professions in the Italian context (Comitato Unico di Garanzia dell’Università degli Studi di Milano, 2017), but we have to point out that women usually have a greater probability of meeting criteria for Post-Traumatic Stress Disorder (Tolin and Foa, 2006; Carpita et al., 2019): this could have had an impact on our results. In addition, the percentage of students collocating themselves in red, orange, or yellow areas reflects the real national situation at the time of our recruitment; it is not the same for the year of attendance: the vast majority of the students attended the first, second, or third year, while only about 13% of our samples came from more senior years. In fact, all the health profession curricula, except for medical and dental health ones, last 3 years, but we have to point out the fact that our sample is composed of a majority of medical students, so we may conclude that our sample is not well balanced. These limitations are due to the convenience method we used for the recruitment, considering the restrictions imposed by the COVID-19 situation. Second, as the pandemic evolves, it is possible that at different times and places, the impact on students could be different from the one that we found. For example, the emotional impact of the pandemic could be different over the different waves (Li et al., 2021b) and in different contexts. Due to the pandemic constant evolution and differences across contexts, the generalizability of our results should be approached carefully.

Third, our survey entirely relies on self-report items and questions. Among the advantages of these psychological instruments, it is necessary to consider also their well-known limitations (Demetriou et al., 2015); moreover, due to setting characteristics and to avoid the cognitive burden, we chose to investigate some constructs with a single-item question and to focus only on the mentioned variables, avoiding collecting other data (as data about the location of Institutions and Universities in which students are enrolled).

Fourth, our study has a cross-sectional design. We could not compare our data collected during the pandemic with psychological symptoms and distress before the pandemic. Also, we did not collect any information about other risk factors, previous psychiatric history, or psychological vulnerabilities that could have influenced the emotional response and distress during the pandemic period.

For these and the abovementioned reasons, we think that further research is needed to understand more in depth and more precisely psychological distress and emotional conditions of students in health professions in the pandemic context.

## Conclusion

Despite these limitations, to the best of our knowledge, our study is the first investigating psychological health and readiness to fight the pandemic in Italian students in health professions during the second wave of the pandemic.

We found that students in health professions had high levels of psychological symptoms, predicted by stress, loneliness, anger, and perceived control. The readiness to fight the pandemic, instead, was predicted by age, year of attendance, fear for their patients, and perceived control.

We think that our data could be useful to Universities and Institutions now and in similar future situations when making decisions about the academic, clinical, and distance learning activities of students, always considering their impact and the impact of the situation itself on their mental health. The point of view of students about the current situation and the new learning technologies should be carefully considered (Hossain et al., 2019). The majority of the literature focused on the involvement of patients in care (Monzani et al., 2020); in our point of view, it may be useful to explore also the readiness of health professional students to be involved in the care process during these situations. Moreover, the readiness of students to fight the pandemic should be considered, together with other numerous factors, in the complex and delicate decision making (Miller et al., 2020; Riva et al., 2020) about the appropriate role that students in health professions should have in pandemic contexts.

## Data Availability Statement

The raw data supporting the conclusions of this article will be made available by the authors, upon reasonable requests.

## Ethics Statement

The studies involving human participants were reviewed and approved by the European Institute of Oncology Ethics Committee – (IEO 1248 – RE 2872). The patients/participants provided their written informed consent to participate in this study.

## Author Contributions

GDM, LV, and GM planned and conducted the study. GDM, LV, GM, and DM drafted the manuscript. All authors contributed to the article and approved the submitted version.

## Conflict of Interest

The authors declare that the research was conducted in the absence of any commercial or financial relationships that could be construed as a potential conflict of interest.

## Publisher’s Note

All claims expressed in this article are solely those of the authors and do not necessarily represent those of their affiliated organizations, or those of the publisher, the editors and the reviewers. Any product that may be evaluated in this article, or claim that may be made by its manufacturer, is not guaranteed or endorsed by the publisher.
