# Supplementary material for: After the Pandemic: The Future of Italian Medicine. The Psychological Impact of COVID-19 on Medical and Other Healthcare-Related Degrees Students
Source: Front Psychol. 2021 Nov 4;12:648419. doi: 10.3389/fpsyg.2021.648419 (PMC8600185; doi:10.3389/fpsyg.2021.648419)
Supplement: Supplementary file 1 [file Table_1.DOCX]

Visual Analog Scale

| Pensando a questa seconda situazione di emergenza volta alla lotta contro il COVID – 19, indica sulla scala sottostante quanto (da 0 a 100) ogni frase rispecchia la tua situazione | Thinking about this second wave of the pandemic, indicate on the scale below how much (from 0 to 100) each sentence reflects your situation |
| --- | --- |
| Ti senti stressato | You are feeling stressed |
| Hai paura per te stesso | You are feeling afraid for yourself |
| Hai paura per i famigliari/persone che vivono con te | You are feeling afraid for your family members/cohabitants |
| Senti di poter controllare la situazione nella vita quotidiana | You are feeling in control of the situation in everyday life |
| Ti senti arrabbiato | You are feeling angry |
| Ti senti solo | You are feeling lonely |
| Ti senti abbandonato dalla tua istituzione formativa/università | You are feeling abandoned by your Educational Institution/University |
| Ti senti preoccupato che la pandemia ritarderà la tua laurea | You are worried that the pandemic will delay your graduation |
| Dopo la pandemia ti sei pentito rispetto alla scelta del tuo corso di laurea | After the pandemic, you regretted choosing your degree program |
| Rispetti le regole imposte per contrastare l’attuale emergenza sanitaria | You respect the rules to counter the current health emergency |
| Pensi che il comportamento da te adottato possa proteggere te stesso e chi ti sta intorno? | Do you think your behavior is protecting yourself and those around you? |
| Il quadro generale della regione in cui ti trovi influisce sul tuo livello di stress | The overall situation of the region you are affects your stress level |
| Hai paura per i tuoi pazienti (se stai svolgendo attività in ospedale) | You are afraid for your patients (if you are working in the hospital) |
| Se hai scelto una carriera in ambito sanitario, quanto ti sentiresti pronto ad affrontare il quadro pandemico odierno | If you chose a career in healthcare, how ready would you feel to face current pandemic situation |
